# Sensitivity of polymerase chain reaction in the detection of rat meat adulteration of beef meatballs in Indonesia

**DOI:** 10.14202/vetworld.2020.905-908

**Published:** 2020-05-15

**Authors:** G. Y. Suryawan, I. W. Suardana, I. N. Wandia

**Affiliations:** 1 Department of Veterinary Medicine, Faculty of Veterinary Medicine, Udayana University, Jl. PB. Sudirman, Denpasar, Bali, Indonesia; 2 Department of Preventive Veterinary Medicine, Laboratory of Veterinary Public Health, Faculty of Veterinary Medicine, Udayana University, Jl. PB. Sudirman, Denpasar, Bali, Indonesia; 3 Department of Basic Veterinary Medicine, Laboratory of Veterinary Anatomy, Faculty of Veterinary Medicine, Udayana University, Jl. PB. Sudirman, Denpasar, Bali, Indonesia

**Keywords:** beef meatball, food safety, polymerase chain reaction method, public health, rat meat, sensitivity

## Abstract

**Background and Aim:**

Meatballs are a processed product of animal origin that is consumed cooked, usually with chicken, beef, or pork as the main ingredient. Unfortunately, some unscrupulous sellers in Indonesia may adulterate this product with rat meat to decrease production costs. Rat meat in any food is a critical public health issue and is prohibited under Indonesian food safety laws, as well as within Muslim communities. This study aimed to test the sensitivity of the polymerase chain reaction (PCR) method in the detection of rat meat contained in processed, cooked beef meatballs.

**Materials and Methods:**

Beef meatballs were formulated with different concentrations of rat meat. Molecular detection of adulteration was initiated by DNA extraction of each cooked meatball formulation followed by PCR using a specific primer for mitochondrial DNA Cytochrome b gene of rat, which primer sequences, i.e., forward primer: 5’CATGGGGACGAGGACTATACTATG ’3 and reverse primer: 5’GTAGTCCCAATGTAAGGGATAGCTG’3.

**Results:**

Our study showed that the PCR method is sensitive in detecting 5% or greater rat meat adulteration of cooked beef meatballs.

**Conclusion:**

The PCR method can be used to detect most rat meat adulteration of cooked beef meatballs and offers a sensitive and effective means to protect food safety and religious requirements in Indonesia.

## Introduction

Animal protein is an important substance for human health as it contains amino acids that increase the body’s metabolism and energy [1]. According to data from the Department of Livestock and Animal Health, Republic of Indonesia, the average amount of animal protein consumed by people in Indonesia increased by 0.261 kg/capita/year in 2013 to 0.469 kg/capita/year in 2017 [2]. When meat consumption increases, this, unfortunately, can also drive criminal activities such as meat fraud, including the adulteration of meats for human consumption with rat or other illegal or inferior meats. Therefore, to protect food safety and to meet religious requirements, sensitive and effective methods to detect the adulteration of meat are urgently needed [2].

Meatballs are a very popular food in all classes of Indonesian society, partly due to their cheap cost and availability. The basic ingredients include beef, chicken, or pork [3], but have found that some producers have fraudulently mixed other kinds of meat with beef or chicken to decrease production costs and increase profits. Anecdotally, one of famous newspapaer in Indonesia published at jpnn.com reported at Nunukan District, North Kalimantan Province in 2017, as many as three beef meatball samples with codes 20, 21 and 22 contained rat meat. These and other anecdotal accounts have the potential to undermine confidence in food safety in Indonesia and interfere with religious food requirements of the Muslim majority in Indonesia.

The adulteration of meatballs with rat meat also breaches Indonesian food laws [1,4]. However, enforcement of these laws relies on the ability to detect the rat meat accurately. Recently, a molecular technique has been developed in Indonesia as a rapid, sensitive, and accurate method to detect the adulteration of meat with other meats not fit for human consumption [5-7]. Furthermore, the polymerase chain reaction (PCR) or its generation as a specific and sensitive method has been developed by some researchers such as the amplification of 12S rRNA gene by Rodriquez *et al*. [7], PCR-base fingerprinting technique by Saez *et al*. [8], multiplex PCR assay by Ali *et al*. [9], or employing real-time PCR by Widyasari *et al*. [10].

Hence, this study aimed to evaluate the sensitivity of the conventional PCR method in the detection of rat meat adulteration of cooked beef meatballs.

## Materials and Methods

### Ethical approval

The approval from the Institutional Animal Ethics Committee to carry out this study was not required as no live animals were used. The rat meat was collected from other studies with ethics approval as part of carcass disposal. This use of the carcass aligns with the reduction principle of the 3 R’s (Reduction, Replacement, and Refinement).

### Meatball formulation

Beef meatballs using minced sirloin were formulated in the laboratory using the following concentrations of minced rat meat: P0 (0% rat meat – negative control) 0 g rat meat/20 g beef; P1 (1.25% rat meat) 0.25 g rat meat/19.75 g beef; P2: (2.5% rat meat) 0.5 g rat meat/19.5 g beef; P3 (5% rat meat) 1 g rat meat/19 g beef; P4 (10% rat meat) 2 g rat meat/18 g beef; P5 (15% rat meat) 3 g rat meat/17 g beef; P6 (20% rat meat) 4 g rat meat/16 g beef; P7 (25% rat meat) 5 g rat meat/15 g beef, and P+ (100% rat meat – positive control) 20 g rat meat/0 g beef). The formulated beef meatballs were then soaked in boiling water until cooked and ready for further testing.

### DNA isolation

Deoxyribonucleic acid (DNA) isolation was done using the Purelink DNA Mini Kit according to the manufacturer’s procedure with a slight modification [11,12]. Two hundred microliters of each meatball suspension (P0 to P+) were added to 200 μl lysis buffer and 25 μg proteinase K in 1.5 ml tubes. The suspensions were then shaken and incubated in a water bath at 56°C for 15 min followed by the addition of 200 μl ethanol and incubation at room temperature for 5 min. The suspensions were then inserted into a spin column and centrifuged at 10,000 rpm for 1 min. The collection tubes were then replaced, and 500 µl of washing buffer was added on the spin column before it was centrifuged at 10,000 rpm for 1 min. The liquid in the collection tubes was removed before the addition of 500 µl washing buffer and centrifugation at 10,000 rpm for 1 min. Collection tubes were then replaced with new ones and again centrifuged at 10,000 rpm for 1 min. The collection tubes were replaced with 1.5 ml recovery tubes and 40 ul nuclease-free water was added to each before centrifugation at 12,000 rpm for 1 min. Finally, the DNA obtained was stored in -20°C before being used.

### PCR

The PCR program was carried out in 25 μl reaction mix containing 5 μl DNA template (200 ng/μl), 18 μl PCR SuperMix 2×, and 2 μl (20 pmol/μl) of each primer F: 5-CATGTGGGACGAGGACTATACTATG-3 and R: 5-GTAGTCCCAATGTAAGGGATAGCTG-3 [13]. The PCR amplification was then performed by initial DNA denaturation at 95°C for 5 min, followed by 35 cycles that consisted of denaturation at 95°C for 30 s, annealing at 55°C for 30 s, and elongation at 72°C for 1 min. The last PCR stage was extended by 5 min at 72°C. The PCR results were analyzed by electrophoresis using 5 μl of PCR products on 1.5% agarose (Gibco BRL, USA) gel, at 125 volts for 35 min. The gel was then stained with 1% ethidium bromide solution (50 μl/L) and destained with T *ris/borate/EDTA* for 10 min. The gel was visualized using UV transillumination and recorded on a digital camera FE-270 with a 7.1-megapixel lens. Positive results were indicated by the presence of PCR product in 188 base pair (bp) position.

## Results

**Figure-1:**
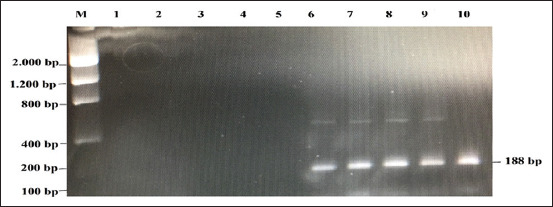
Amplification of the *cyt-b* mitochondrial gene using specific primers in the detection of rat meat in beef meatballs. M: marker 100 bp; 1-5: 100 % of beef; 6-10: 100 % of rat meat.

The PCR amplification detected the mitochondrial DNA (*mtDNA*) cytochrome *b* (*cyt-b*) gene in rat meat using specific primers that showed a positive result, which is characterized by PCR product 188 base pair (bp) (Figure-1).

**Figure-2:**
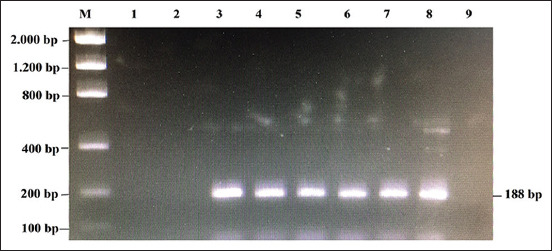
Results of PCR detection in various concentrations of rat meat on 1.5% agarose gel. M: marker 100 bp; 1-8: rat meat concentrations *i.e*. 1.25%; 2.5%; 5%; 10%; 15%; 20%; 25%; and 100%, respectively. 9: 100% of beef as a negative control.

The result of the PCR analysis in Figure-1 shows that the primer used in this study is able to differentiate between cooked beef and rat meat. Based on the power of the primer discrimination, the study was continued by measuring the PCR sensitivity using different concentrations of rat meat. The results of the study are presented in Figure-2.

The data in Figure-2 show that rat meat could be detected using the PCR method as low as at 5% concentration. To test the consistency of this method, the study was repeated 10 times (Table-1).

**Table-1:** The consistency of PCR method in the amplification of *cyt-b* mitochondrial gene of rat meat with various concentrations.

Concentrations of rat meat	Repetitions										Consistency

	1	2	3	4	5	6	7	8	9	10	
1.25%	-	-	-	-	-	-	-	-	-	-	100%
2.5%	-	-	-	-	-	-	-	-	-	-	100%
5%	+	+	+	+	+	+	+	+	+	+	100%
10%	+	+	+	+	+	+	+	+	+	+	100%
15%	+	+	+	+	+	+	+	+	+	+	100%
20%	+	+	+	+	+	+	+	+	+	+	100%
25%	+	+	+	+	+	+	+	+	+	+	100%
K+	-	-	-	-	-	-	-	-	-	-	100%
K -	-	-	-	-	-	-	-	-	-	-	100%

The data in Table-1 demonstrate the consistency of the PCR method in the detection of rat meat at concentrations as low as 5%. This consistency is shown clearly by the PCR products 188 bp in 10 repetitions. The results also consistently show negative results at both 1.25 and 2.5% concentrations of rat meat.

## Discussion

Meatballs are a very popular traditional dish in Indonesia, partly due to their cheap cost and availability. The basic ingredients include beef, chicken, or pork, but some studies have found instances of food fraud, in which the advertised meat is, in fact, a mixture of other kinds of meat [3]. Beef is generally the most expensive meat in Indonesia and thus, the cost of making beef meatballs is higher than for other types of meat. As a result, this has encouraged some sellers to adulterate beef meatballs with inferior or illegal meats to decrease the production cost and maximize profits.

Adulteration means that substances have been added to food that changes its composition and impact its nutritional value [14] and societal acceptance. There is anecdotal evidence that rat meat, which is similar in color and texture to beef, may be added to meatballs, but this has been difficult to prove. It is very important for Indonesia to maintain food safety and confidence in food security. The presence of rat meat in meatballs is a serious food safety risk as well as being *haram* or forbidden for Muslims, who make up 87.18 % of Indonesia’s population [1,15].

The PCR method is a backbone of molecular technology and has been used worldwide to identify adulteration of meat products due to its sensitivity and accuracy. Older methods using the identification of lipids and proteins are considered unreliable due to biochemical changes during processing and cooking. These problems prompted scientists to consider the potential of DNA testing in food safety [14]. The PCR method also needs only a small amount of DNA [8,16]. The use of PCR and its modification as a fast rapid and sensitive method has been demonstrated by several researchers worldwide, as well as in Indonesia. Matsunaga *et al*. [17] from Japan in 1999 applied multiplex PCR to differentiate the origins of six meats, including cattle, pig, chicken, sheep, goat, and horse. The primers were designed to amplify the *cyt-b* of each *mtDNA* of each species. The forward primer amplified the conserved DNA sequence of the different species of the mitochondrial gene, while reverse primers were designed on the specific species of DNA sequences [17]. Srihanto *et al*. [13] from Indonesia in 2015 employed real-time PCR to successfully amplify rat DNA from processed meatballs using specific primers of *cyt-b* of *mtDNA*.

Hence, *cyt-b* is known as one part of the cytochrome that involves the genetic transport of mitochondria. The *cyt-b* contains eight helical transmembranes connected by intramembranous or extramembranous domains. The *cyt-b* is encoded by *mtDNA*, which is commonly used to determine the phylogenetic relationships among organisms due to its sequence variability. It is also considered to be most useful in determining the relationships between families and genera. Furthermore, some comparative studies involving *cyt-b* have been used in new classification schemes and to assign newly described species or genus, as well as to gain a deeper understanding of evolutionary relationships [18].

In addition, in our study, the conventional PCR method with a specific primer has been successfully used to amplify *cyt-b* gene from rat meat at various levels of adulteration. The beef meatballs adulterated with rat meat were detected to as low as 5% concentration of rat meat, over 10 PCR repetitions. Furthermore, the PCR also appears to be accurate at the lower concentrations of 1.25% and 2.5% rat meat, as these were not detected in any of the PCR runs. Our study also confirmed that the PCR method was successful in detecting rat meat adulteration in products that had undergone processing and cooking. Thus, our results support the superiority of the conventional PCR method to detect rat meat adulteration of cooked beef meatballs on the basis of its sensitivity, efficiency, especially for high scales of production and accuracy. Of course, the success of this method also depends on several factors, including specific primary use, good reagent, sample storage, and no *DNAse* contamination [6,19].

Furthermore, additional confirmation of PCR products can be obtained by the sequencing of DNA amplicons, restriction fragment length polymorphism analysis (PCR-RFLP), and real-time PCR and single-strand conformation polymorphism analysis (PCR-SSCP). The techniques used to obtain the product include amplified fragment length polymorphism analysis (AFLP), analysis of inter-simple sequence repeat polymorphism, analysis of short tandem repeat polymorphisms, and amplification of multiple templates during a single PCR reaction (Multiplex PCR) [14].

Random amplified polymorphic DNA (RAPD) was the first PCR-based technique and is currently the simplest one. Randomly chosen short primers (about 10 bp) are used to amplify DNA in the genome. This method makes it possible to obtain a larger number of products by reproducing unspecified fragments of template DNA [14], as well as the use of arbitrary primed PCR methods which has the same principle with RAPD that was used by Suardana *et al*. [20] in identification of the diversity of *Escherichia coli* O157:H7.

## Conclusion

The PCR method is a very useful tool to improve food safety and security in Indonesia by providing authorities with a sensitive and cost-effective means to detect 5% or greater rat meat adulteration of cooked beef meatballs. This is also very important to Indonesia’s Muslim majority as rat meat is haram or forbidden.

## Authors’ Contributions

GYS, ISW, and INW conceived and designed the experiments. GYS performed the PCR. All authors read and approved the final manuscript.
